# Influences of Land Use/Cover Types on Nitrous Oxide Emissions during Freeze-Thaw Periods from Waterlogged Soils in Inner Mongolia

**DOI:** 10.1371/journal.pone.0139316

**Published:** 2015-09-25

**Authors:** Zedong Lu, Rui Du, Pengrui Du, Saisai Qin, Zongmin Liang, Ziming Li, Yaling Wang, Yanfen Wang

**Affiliations:** University of Chinese Academy of Science, Beijing 100049, China; Institute of Tibetan Plateau Research, CHINA

## Abstract

Nitrous oxide emissions during freeze/thaw periods contribute significantly to annual soil N_2_O emissions budgets in middle- and high-latitude areas; however, the freeze/thaw-related N_2_O emissions from waterlogged soils have hardly been studied in the Hulunber Grassland, Inner Mongolia. For this study, the effects of changes in land use/cover types on N_2_O emissions during freeze–thaw cycles were investigated to more accurately quantify the annual N_2_O emissions from grasslands. Soil cores from six sites were incubated at varying temperature (ranging from −15 to 10°C) to simulate freeze–thaw cycles. N_2_O production rates were low in all soil cores during freezing periods, but increased markedly after soil thawed. Mean rates of N_2_O production differed by vegetation type, and followed the sequence: *Leymus chinensis* (LC) and *Artemisia tanacetifolia* (AT) steppes > LC steppes ≥ *Stipa baicalensis* (SB) steppes. Land use types (mowing and grazing) had differing effects on freeze/thaw-related N_2_O production. Grazing significantly reduced N_2_O production by 36.8%, while mowing enhanced production. The production of N_2_O was related to the rate at which grassland was mowed, in the order: triennially (M3) > once annually (M1) ≥ unmown (UM). Compared with the UM control plot, the M3 and M1 mowing regimes enhanced N_2_O production by 57.9% and 13.0% respectively. The results of in situ year-round measurements showed that large amounts of N_2_O were emitted during the freeze–thaw period, and that annual mean fluxes of N_2_O were 9.21 μg N_2_O-N m^-2^ h^-1^ (ungrazed steppe) and 6.54 μg N_2_O-N m^-2^ h^-1^ (grazed steppe). Our results further the understanding of freeze/thaw events as enhancing N_2_O production, and confirm that different land use/cover types should be differentiated rather than presumed to be equivalent, regarding nitrous oxide emission. Even so, further research involving multi-year and intensive measurements of N_2_O emission is still needed.

## Introduction

Nitrous oxide (N_2_O) contributes significantly to global warming [1] and also destroys stratospheric ozone [2]. Significant sources of N_2_O are found in grasslands [3], which are an important component of global terrestrial ecosystems and cover about 25% of the global land surface [4]. Even minor alterations to radiatively active trace gases between grassland ecosystems and the atmosphere can be significant for global atmospheric budgets [5].

The human practices of mowing and grazing are important in the semi-arid grasslands of Inner Mongolia. The effects of grazing vary with grazing intensity [6] (categorized as light, moderate, or heavy). Previous studies have shown that light and moderate grazing intensities stimulate the growth of grasses and grassland productivity [7, 8]. Grazing compacts soil and increases soil bulk density by animal trampling [9], which reduces permeate–water flux and thus leads to reduced soil water content [10, 11]. Moreover, grazing removes much aboveground biomass, which allows more daylight at the soil surface and increases surface temperature. High temperature can accelerate decomposition of SOC [12]. Although grazing reduces grass residue returning to soil, animal excrement (dung and urine) input could reduce loss of nutrients by runoff [13] and enhance the rate of N cycling [14]. Grazing management also affects soil microorganisms [15, 16]. In combination, these effects strongly influence N_2_O emissions. Recent studies reported that grazing decreased N_2_O emission because the effects of grazing on inorganic nitrogen, soil moisture, and soil microbes were greater than those on N cycling [17]. Mowing inhibits surface litter accumulation [18, 19] and alters plants’ access to light [20], soil surface temperature, soil moisture [21], and microbial growth [21, 22]. To date, the underlying mechanisms and the effects of mowing on greenhouse gas (GHG) emissions remain uncertain. Previous studies suggested that mowing facilitated CH_4_ uptake in grassland because of reduction in soil inorganic N [23], and weakened N_2_O emission through its effect on vegetation types and some soil properties [24]. Land cover types also affect GHG fluxes because different litter quality is a key factor regulating decomposition and release of labile nitrogen and carbon compounds [25, 26]. Matson et al. [27] and Corre et al. [28] noted the dynamics of soil organic matter (C and N) cycling among land use/cover types as a consequence of environmental and soil characteristics [27, 28].

N_2_O emissions from soils mainly derive from microbial nitrification and denitrification, even when the soil temperature is near freezing [29, 30, 31, 32]. To date, large episodic emissions of N_2_O have been confirmed during the process of soil thawing [33, 34, 35]. The processes by which N_2_O production increases during soil thawing have also been discussed. Early studies reported that N_2_O was produced in unfrozen subsoil and physically released from the soil surface when the frozen soil thawed [36, 37]. Recently, most studies have suggested that emissions of N_2_O derive from enhanced biological activity via nutrient oversupply [38, 39, 40]. N_2_O emissions during the winter period can account for 0–93% of annual emissions [29], and are related to the freeze/thaw cycles (FTCs) of soil. However, FTCs are generally limited to a few days in winter, and depend on specific site and weather conditions; for these reasons, there is uncertainty about N_2_O emissions during winter, which makes it difficult to predict annual N_2_O emissions accurately. In situ measurements from various ecosystems revealed that N_2_O emissions during the freeze–thaw period can account for a large proportion of annual N_2_O emissions from field ecosystems, tundra, and boreal forest ecosystems [40, 41, 42, 43, 44]. In China, previous studies of greenhouse gas (GHG) emissions during the freeze–thaw period have been concentrated in the Qinghai–Tibet Plateau, Xilin River catchment, and Sanjiang Plain [5, 17, 25, 45, 46, 47, 48].

The Hulunber meadow-steppe of Inner Mongolia covers an area of about 1,419,000 km^2^ in the eastern part of the Eurasian grassland biome, and is a typical and highly important meadow-steppe grassland in China. Moreover, the Hulunber grasslands have special ecological characteristics related to climate and geography, with a gradual shift in vegetation types from meadow steppes in the east to typical steppes in the west. Most previous N_2_O measurements at this site were conducted during the growing season [49], and N_2_O fluxes in this type of grassland during the freeze–thaw period have not been investigated previously. Establishing the percentage of N_2_O emissions that derives from the freeze–thaw cycle might be helpful for reliably evaluating annual N_2_O emissions. The most common practices in Hulunber grasslands are mowing for hay, and grazing by livestock. The FTC-induced N_2_O fluxes could be expected to vary under the influence of land use/cover types in waterlogged soils, although this is poorly studied. Consequently, there is an urgent need to clarify the phenomenon that occurs in waterlogged soils during FTCs, in order to better understand gaseous N losses from Inner Mongolia grasslands.

To better understand the potential production of N_2_O in this field during the FTCs, we conducted a laboratory incubation study in which soil was kept waterlogged during the entire winter and into early spring. The objectives of present study were: (1) to assess the effects of land use (steppe, mown steppe, and grazed steppe) and land cover (*Leymus chinensis*, *Stipa baicalensis*, and *Artemisia tanacetifolia*) types on N_2_O production from soil in the Hulunber Grassland, subjected to several successive FTCs in laboratory conditions; and (2) to quantify N_2_O fluxes during a freeze–thaw event from the year-round field measurements.

## Materials and Methods

### Study site

The experimental grassland site was located around the Hulunber Grassland Ecosystem Research Station of the Chinese Academy of Agricultural Sciences in Inner Mongolia, China (49°10′ N, 120°03′ E; 628 m.a.s.l). Permission to use each site was issued by the chief of station, Professor Xiaoping Xin, and the field studies did not involve endangered or protected species. The regional climate is semi-arid, with a frost-free period of 95–110 days, from early May to early September. The winter season is cold and dry, lasting approximately from October to April of the following year. Annual mean air temperature is −2 to −1°C. The maximum and minimum mean monthly temperatures occurred in July (21°C) and January (−26°C), respectively. Annual mean precipitation is 400 mm with large inter-annual variation (150–550 mm) and most rain falling between June and August. The soil in this region was subjected to several episodes of repeated FTCs. The soil started to freeze in late October, and started to thaw in April. The general soil properties at each study site are shown in **Table 1**.

**Table 1:** General soil properties of the soil sampling sites^a^.

Plot code	BD (g cm^-3^)	pH (H_2_O)	SOM (g kg^-1^)	Total N (g kg^-1^)	NH_4_^+^-N (mg kg^-1^)	NO_3_^−^-N (mg kg^-1^)	Land cover type	Soil type
UM	1.16	7.1	55.13	2.71	4.45	8.32	*Carex tristachya*	Chernozem Soil
M1	1.13	7.5	51.86	2.45	3.63	2.90	*Leymus chinensis* and *Artemisia tanacetifolia*	Chernozem Soil
M3	1.12	7.9	44.87	1.86	4.34	11.15	*Leymus chinensis* and *Artemisia tanacetifolia*	Chernozem Soil
LUG	1.23	6.5	59.54	2.98	0.81	26.57	*Leymus chinensis*	Chestnut Soil
SUG	1.13	6.8	69.68	3.03	0.85	22.86	*Stipa baicalensis*	Dark Chestnut Soil
SG	1.27	6.6	60.11	2.97	0.97	23.50	*Stipa baicalensis*	Dark Chestnut Soil

^a^Abbreviations are as follows: BD, bulk density; SOM, soil organic matter; UM, un-mowed grassland; M1, mowed once annually; M3, mowed once triennially; LUG, *Leymus chinensis* grassland; SUG, *Stipa baicalensis* grassland; SG, *Stipa baicalensis* grazed grassland

Soil columns were taken at three non-adjacent grassland sites (GS1–3). The first study site (GS1) covered 33 ha, and was dominated by *Leymus chinensis* (*LC*). The second study site (GS2) had an area of 32 ha and was characterized by *Stipa baicalensis* (*SB*). At both sites, enclosures were established in 2007 to prevent animal grazing (Plot LUG at GS1; Plot SUG at GS2); while the grassland close to the enclosed area (control) was grazed during the plant growing season (Plot LG at GS1; Plot SG at GS2) [49]. It is regrettable that the effect of grazing at Plot LG was not included in our present study. This was prevented by the drift of snow into the low-lying LG plot, to a depth of about 40 cm. The third study site (GS3) was dominated by *LC* and *Artemisia tanacetifolia* (*AT*), and had an area of 34 ha. The site has been fenced since 2005 to investigate the effects of mowing on grassland ecosystems. On separate plots there, the grass was mowed once annually (Plot M1) or once triennially (Plot M3). The control plot was not mowed (Plot UM). Since August of 2009, the grass there has been mowed by machine, to a height of 5 cm above the surface.

### Laboratory experiment

#### Soil sampling

Intact soil samples were taken at plots UM, M1, and M3 in May 2012, and at plots LUG, SG, and SUG in May 2013. In order to minimize soil spatial heterogeneity, soils were collected near the static chamber. Each soil sample (approximately 25 × 25 × 20 cm) was dug up after sweeping away the biomass covering the surface, and sent to the laboratory for further treatment. Then, each soil sample was evenly divided into five depth-layers (0–3, 3–6, 6–9, 9–12, and 12–15 cm) using a slicing knife. Next, each depth-layer was carefully cut into six pairs of soil columns (3 cm deep, 5 cm in diameter) using a homemade stainless-steel cylindrical ring knife (5 cm diameter). Soil column samples were immediately placed in zip-lock bags and stored at −20°C in the laboratory until used for incubation (three replicates for each treatment) and soil property analysis (three replicates for each treatment).

#### Experimental setup

**Fig 1:**
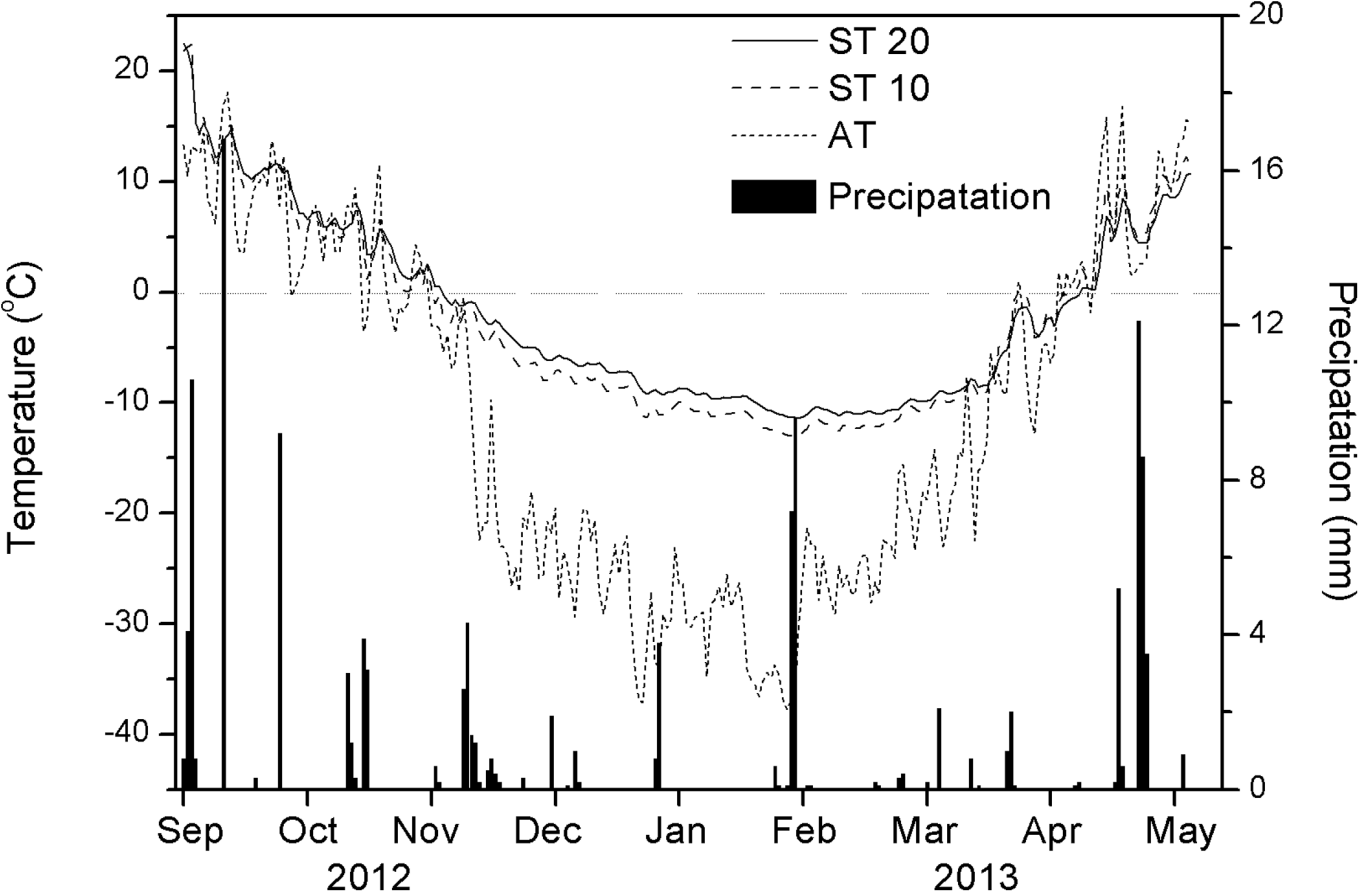
Conditions at SG site from September 2012 to May 2013: Daily mean air temperature (AT), daily mean precipitation, and soil temperature at depths of 10 cm depth (ST10) and 20 cm (ST20).

The FTC experiments were performed in 360 ml flasks. Tap water (directly extracted local groundwater) was used to just flood the top of the soil, to simulate waterlogged conditions in the field. The flask still had sufficient headspace remaining at the top of the soil column for gas sampling. Then, each flask was sealed using an airtight cap and placed in a freezer to simulate a series of FTCs. The soil temperature at depths of 10 and 20 cm at the study sites ranged from about −15 to 10°C throughout the winter (**Fig 1**), thus the effects of temperature were examined at various temperatures (−15, −10, −5, 5, or 10°C), followed by laboratory incubation. The total duration of the FTC experiment was about eight weeks and the incubation temperature cycle was: −15 to 5; 5 to −10; −10 to 5; 5 to −5; −5 to 5; 5 to −5; and −5 to 10°C. For example, during the first FTC, the soil was frozen for one week at −15°C and thawed to 5°C for one week. During the second FTC, the temperature was lowered from 5 to −10°C for one week, then warmed to 5°C. In the subsequent cycles, the temperature was changed in the same way, to reach the target temperatures listed above.

#### Gas sampling and analysis

Three 2-ml gas samples were taken from the headspace of each flask with 60 mL plastic syringes fitted with three-way stopcocks, at 12 h, 24 h, 3 days, and 7 days. After 24 h, the lids were removed until 10 h prior to the collection of gas samples. Each gas sample was analyzed immediately via gas chromatography (Agilent 7890A, Agilent Technologies, USA). N_2_O and CO_2_ fluxes from waterlogged soils in laboratory incubation experiments (*f*_incubation_) and in filed measurements (*f*_field_), and cumulative N_2_O emissions (*X*_n_) and mean N_2_O fluxes (*F*) were calculated using the following equations described by Du et al. [50]:

$$f_{\text{incubation}}=\frac{\text{d}m}{M_{\text{soil}}\text{d}t}=\frac{\rho \upsilon \text{d}c}{M_{\text{soil}}\text{d}t},$$ (1)

$$f_{\text{field}}=\frac{\text{d}c}{\text{d}t}\frac{M_{\text{gas}}}{V_{0}}\frac{P}{P_{0}}\frac{T_{0}}{T}H,$$ (2)

$$X_{\text{n}}=\frac{f_{\text{n}}+f_{\text{n}-1}}{2}\times (D_{\text{n}}-D_{\text{n}-1}),$$ (3)

$$F=\frac{\left(X_{1}+X_{2}+\cdots +X_{\text{n}}\right)}{\left(D_{\text{f}}-D_{0}\right)}$$ (4)

where *ρ* is the density of the gas, d*c*/d*t* is the variation ration of the gas concentration, *M*_soil_ is the mass of the soil, *v* is the volume of headspace in the flask, *M*_gas_ is the mole mass of gas, *P* is the atmospheric pressure, *T* is the absolute temperature, and *H* is the height of chamber above the surface. *V*_0_, *P*_0_, and *T*_0_ are volume, air absolute temperature, and pressure at standard conditions. *f*_n_ is the N_2_O flux measured at the end of the specific period, *f*_n-1_ is the N_2_O flux measured at the end of the previous specific period. *D*_n_ is the last day of the specific period, *D*_n-1_ is the last day of the previous period, *D*_0_ is the first day of the experiment, and *D*_f_ is the final day of the entire sampling period.

#### Measurement of soil chemical properties

Soil NH_4_^+^ and NO_3_^−^ content, soil organic matter (SOM), and total nitrogen (TN) from five different depths were analyzed at the start and end of each experiment. Soil NH_4_^+^ and NO_3_^−^ were measured photometrically using a photometric flow-injection analyzer (FIA Star 5000, Foss Inc., Hillerød, Denmark) after extraction from the soil with KCl suspension (1:2 w/w 1M KCl) for 1 h. Total N content was analyzed via an auto-analyzer (Foss Inc., Hillerød, Denmark) using the Kjeldahl method. Organic matter content was determined by the soil bath-K_2_CrO_7_ titration method [51]. Soil bulk density was determined gravimetrically, and pH was measured with a glass electrode (F-22, Horiba, Kyoto, Japan) in a water solution using fresh soil (1:2.5 w/w) before incubation.

### Field measurements

#### Gas sampling

At each site, subplots (50 × 50 cm, four replicates for each treatment) were established to simultaneously observe N_2_O emission along an installed boardwalk. Gas samples were collected using the static chamber method. The static chamber was made of stainless steel and equipped with a fan (10 cm diameter) installed on the top wall of each chamber in order to ensure complete gas mixing. A square box (without a top and bottom; 0.5 m long × 0.5 m wide × 0.1 m high) was inserted directly into the soil to a depth of 10 cm, and the box cover (without a bottom; 0.5 m long × 0.5 m wide × 0.5 m high) was placed on top. A white adiabatic cover was added to the outside of the box cover to reduce the effect of direct radiative heating during sampling. The chamber was then closed and five gas samples were collected at 0, 10, 20, 30, and 40 min using plastic syringes fitted with three-way stop-cocks. The gas samples were analyzed within 24 h using a gas chromatograph (Agilent 7890A, Agilent Technologies, USA). The gas chromatograph (GC) was equipped with an electron capture detector and a flame ionization detector, for N_2_O and CO_2_ analysis, respectively, and the configurations were the same as those reported by Wang and Wang [52]. Flux measurements were taken once or twice per week during the growing season, and once or twice per month during the non-growing season.

#### Measurement of environmental factors

Data on soil temperature at depths of 10 and 20 cm were recorded every minute and saved every five minutes, by an automated measuring system (Hobo Micro Station Data Logger, H21-002, USA). Climatic data in the form of daily precipitation and air temperature were obtained from the local meteorological station at National Hulunber Grassland Ecosystem Observation and Research Station.

### Statistical analysis

Statistical analysis used SPSS 12.0 (SPSS Inc., Chicago, USA), and graphs were created using Origin 8.0 (Origin Lab Corporation, USA). The NH_4_^+^, NO_3_^−^, SOM, and TN contents, measured before freezing and at the end of thawing, were compared using paired *t*-tests. Significant differences in gas flux, between different land use/cover types and FTCs cycles, were determined by repeated-measures ANOVA. All statistical tests were performed at a significance level of level of α = 0.05.

## Results and Discussion

### Air temperature, soil temperature, precipitation conditions

From October 2012 to May 2013, the total number of snow-days was 49, and total precipitation was 87.6 mm, with higher monthly precipitation in November and April. The relatively heavy precipitation and snow resulted in saturated soil conditions. The daily air temperature changed very rapidly at the onset of freezing and thawing events. For example, the daily mean air temperature fell by 13.8°C (from 1 to −12.8°C) within five days, then increased by 17.7°C (from −1.9 to 15.8°C) three days later in April 2013 (**Fig 1**).

The daily mean air temperature dropped below 0°C on 2 November 2012 and rose above 0°C on 7 April 2013. The daily mean soil temperature at 10 cm depth dropped below 0°C on 3 November 2012 and remained below zero until around 11 April 2013 (**Fig 1**). The soil temperature at 20 cm depth dropped below 0°C on 5 November 2012 and remained below zero until around 13 April 2013. After this date, the soil temperature remained at approximately 0°C for about two days and then increased to 10°C (**Fig 1**). Hence, topsoil generally started to freeze in early November and thawed in April, and the soil surface was subjected to seasonal FTCs during April.

Each full FTC could be divided into four periods: progressive freezing, completely frozen, progressive thawing, and completely thawed [47]. From 16 October to 2 November 2012, the soil was in a progressive freezing period with several diurnal freeze–thaw phases. From 3 November 2012 to 27 March 2013, the soil was in a completely frozen period. From 28 March to 16 April 2013, the soil was in a progressive thawing period with seasonal freeze–thaw phases.

### Soil TN, SOM, NH_4_^+^-N, and NO_3_^-_^N concentrations

**Fig 2:**
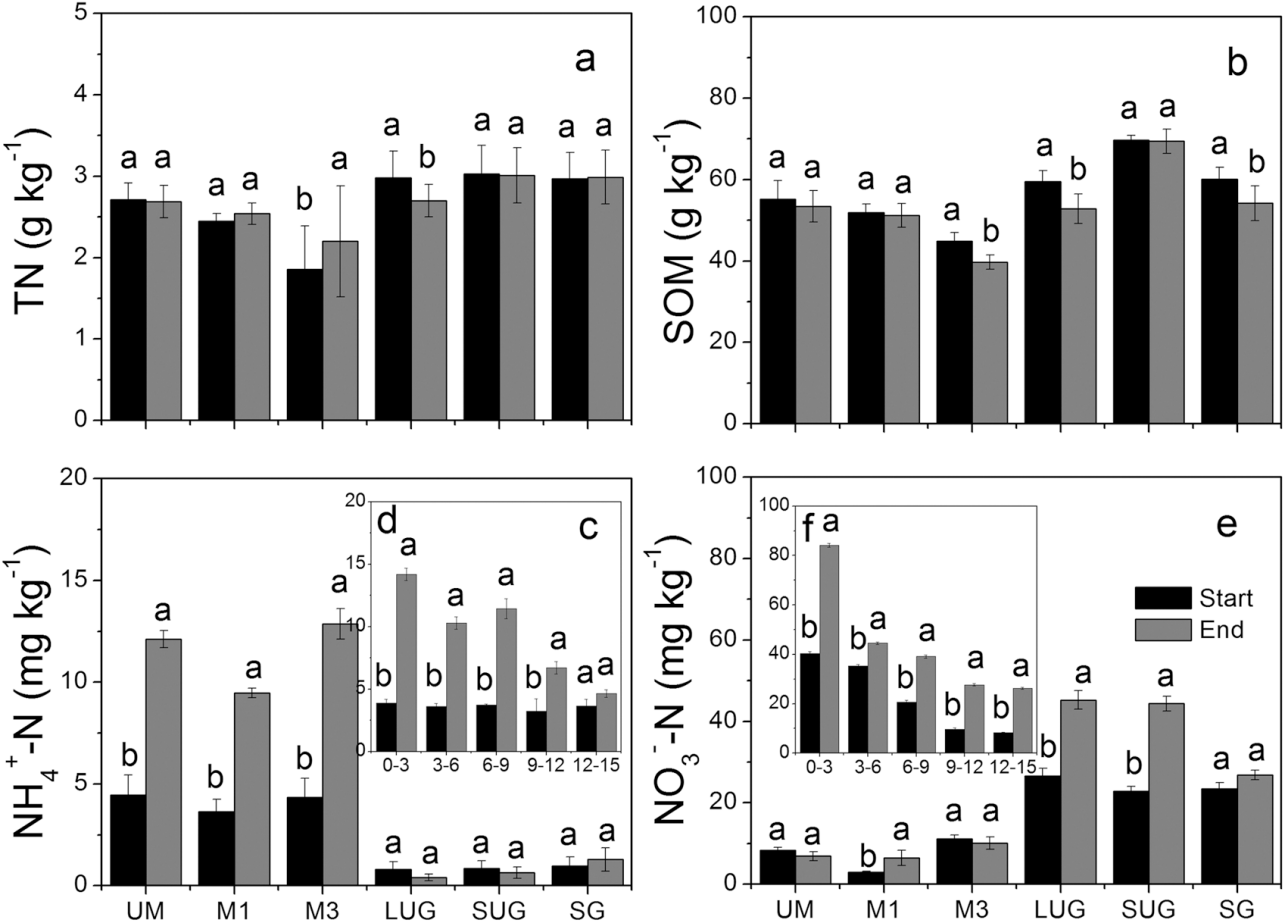
Soil conditions at the start and end of the freeze/thaw incubation experiments: Average total nitrogen (TN) (a), Soil organic matter (SOM) (b), NH_4_^+^ (c), NH_4_^+^ content at different soil depths for M1 (d), and NO_3_^−^ (e), NO_3_^−^ content at different soil depths for SUG (f). The vertical bars indicate standard errors of three replicates.

The soil total nitrogen (TN), soil organic matter (SOM), NH_4_^+^-N, and NO_3_^−^-N concentrations were measured at the beginning and end of entire FTCs (**Fig 2**). TN and SOM concentrations decreased with soil depth (data not shown). Average soil TN ranged from 1.86 to 3.03 g kg^-1^ with no significant differences between the beginning and end of the experiment except in M3 and LUG (**Fig 2A**). Organic matter that derived from the litter layer has been suggested as a source of nutrients and energy for soil microbes [53, 54]. SOM concentration decreased after incubation, with an average reduction rate of 0.06 g kg^-1^ d^-1^ (**Fig 2B**).

The soil NH_4_^+^-N concentration was almost the same at different depths (data not shown) before incubation, but was higher at the end of the experiment, and was remarkably high at plots UM, M1, and M3 (**Fig 2C**). For example, at the beginning of the experiment the NH_4_^+^-N concentration in soil from M1 was 3.63 mg kg^-1^; after the experiment, the NH_4_^+^-N concentration was 9.46 mg kg^-1^, and decreased with soil depth: 14.2 to 3.22 mg kg^-1^ (**Fig 2D**). Soil NO_3_^−^-N content decreased with soil depth at the beginning (data not shown); after incubation, NO_3_^−^-N content was higher than at the start of experiment, especially at plots LUG and SUG (**Fig 2E**).For example, the NO_3_^−^-N concentration in soil from site SUG increased from 22.86 mg kg^-1^ to 44.37 mg kg^-1^ during the experiment. Here also, the NO_3_^−^-N concentration decreased with soil depth, from 84.17 to 8.25 mg kg^-1^ (**Fig 2F**). Zancarini et al noted that a large diversity of soil microbes was able to mineralize soil organic matter and nitrogen from organic nitrogen [55]. The results here revealed that freeze–thaw resulted in increased concentrations of NH_4_^+^-N and NO_3_^−^-N to some extent, with a soil net nitrogen mineralization rate of 0.20 mg kg^-1^ d^-1^. The reduction of SOM concentration and increase of inorganic N concentration (NH_4_^+^-N and NO_3_^−^-N) suggest that microbial activity might have played an important part during freeze–thaw periods, i.e., ammonification and nitrification. The findings also may be caused by release from lysed soil microbes or destroyed soil.

### Soil N_2_O production from laboratory incubation

**Fig 3:**
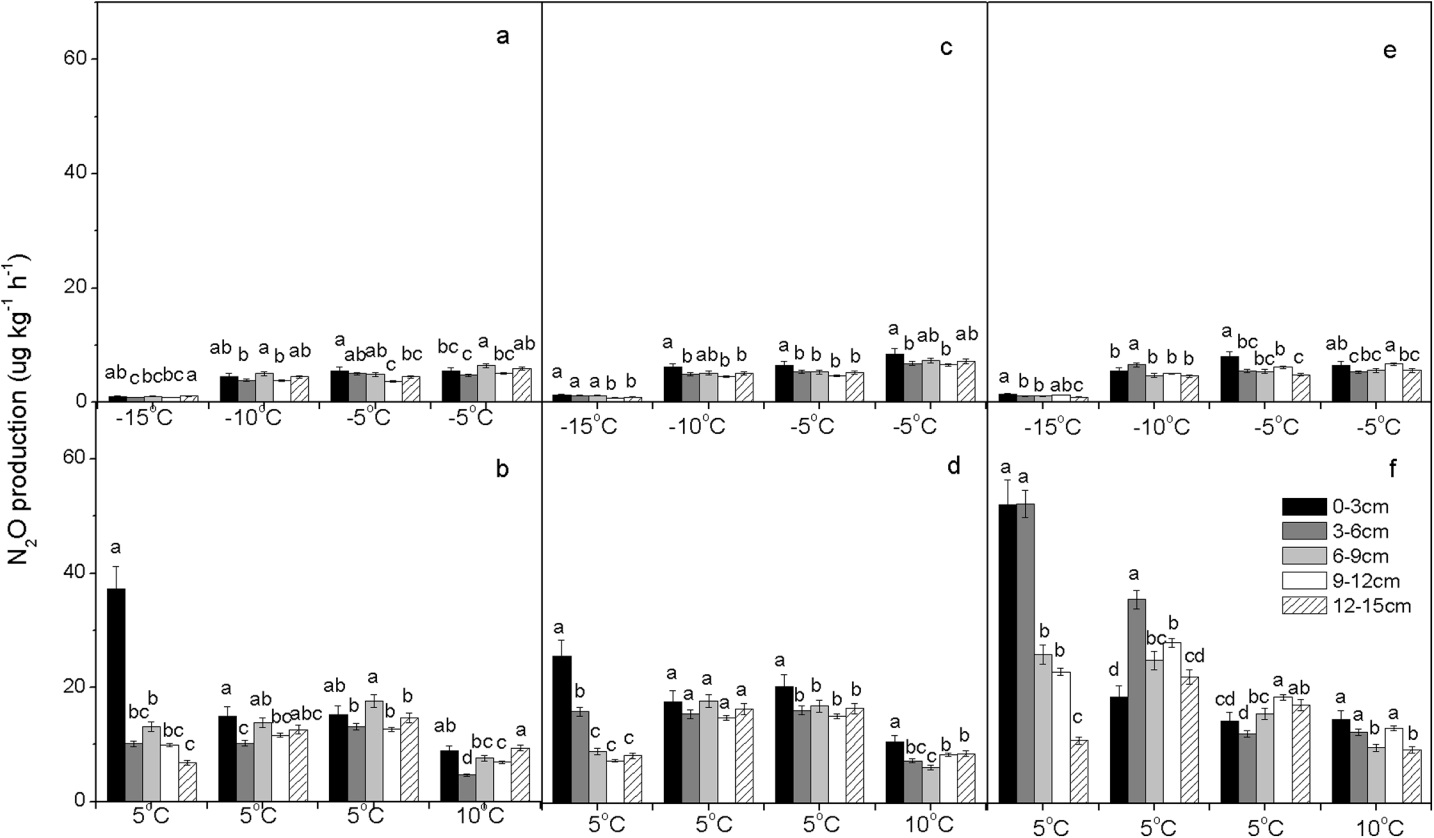
Mean N_2_O production rates (μg kg^-1^ h^-1^) along the profile (0–15 cm) of soil cores: From UM (a, b), M1 (c, d), and M3 (e, f) over the entire incubation period (n = 3); Freezing periods: a, c, e; Thawing periods: b, d, f. Lowercase letters indicate significant differences (P < 0.05) between samples from different soil depths.

**Fig 4:**
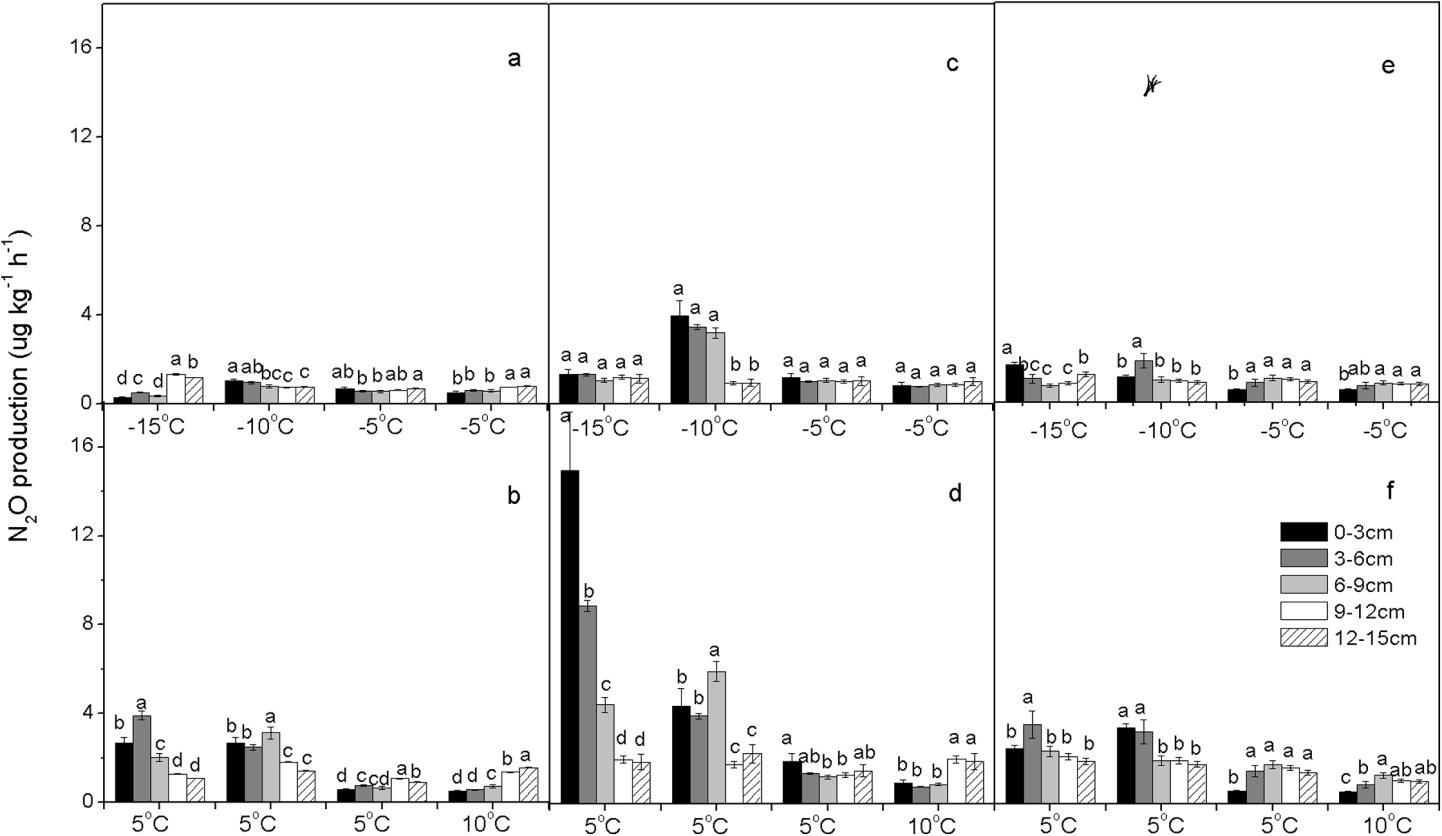
Mean N_2_O production rates (μg kg^-1^ h^-1^) along the soil profile (0–15 cm) of undisturbed soil cores: From LUG (a, b), SUG (c, d), and SG (e, f) during the entire incubation period (n = 3). Freezing periods: a, c, e; Thawing periods: b, d, f. Lowercase letters indicate significant differences (P < 0.05) between samples from different soil depths.

The dynamics of N_2_O production of soil cores from each land use/cover type exposed to experimental FTCs are shown in **Figs 3 and 4**. The biological processes showed positive reactions to temperature change within a range [56], and CO_2_ production rates reflected the activity of all microbes in our incubation study because soil CO_2_ emissions were solely from microbial respiration (**S1 and S2 Figs**). CO_2_ production remained at a low level in the frozen soil. When the soil temperature increased from sub-zero to above zero during thawing, the CO_2_ production rate was immediately enhanced, although the increase was generally less in successive FTCs. For example, in soil from M3, the CO_2_ production rate increased (from 0.01–0.31 to 0.03–0.85 μg g^-1^ h^-1^) soon after the first thaw started (**S1E and S1F Fig**), whereas this effect was very weak in the soil from plots SUG, SG, and LUG (**S2 Fig**).

From **Figs 3 and 4**, it can be seen that large amounts of N_2_O were emitted directly from thawed soil. At −15°C, N_2_O production rates were very low from all soils but never reached zero, and increased significantly at 5°C. For example, the N_2_O production rate was characterized by rapid increase from 2 to 50 μg kg^-1^ h^-1^ during the thawing period, followed by a rapid decline to 10 μg kg^-1^ h^-1^ two days later, when the temperature dropped from 5 to −10°C during a freezing period at M3 (**Fig 3E and 3F**). The variation of N_2_O production rates in each soil layer revealed a similar pattern: In all of the soils, N_2_O production was generally low prior to thawing, and the highest soil N_2_O production was generally observed during soil-thawing periods (**S3 Fig**). However, after several FTCs, soil N_2_O production declined sharply and remained almost constant because of limited substrate availability. This was in line with data analysis of CO_2_ production rates, and was further supported by a significant correlation between CO_2_ and N_2_O production rates (P < 0.01) (**S4 Fig**). As soil temperature increased, the intensification of microbiological activity contributed to increase in CO_2_. This suggests that the elevated N_2_O production resulted in part from biological activity.

Large emissions of N_2_O have been recorded from agricultural soils and grasslands, but data from most previous studies were restricted to thawing periods [25, 57, 58]. Only a few studies have reported that constant N_2_O emission occurred during freezing periods [59, 60]. In this laboratory study, we clearly showed that N_2_O production occurred during both freezing and thawing periods. The N_2_O production (from 0.3 to 5 μg kg^-1^ h^-1^) was measurable for several days in frozen soil at −15°C at both experimental sites (**Figs 3 and 4**). This finding supports those of Neilson et al. [34] and Müller et al. [60], who reported that N_2_O emissions were observed even when soil was frozen. Bremner and Zantua [61] found that enzyme activity occurred in soil at temperatures as low as −20°C [61]. This indicated that the microbial community was still active at −15°C, even deeper in the soil than in our laboratory experiments. Because the air temperature fell much lower in our work (−37.8°C) **(Fig 1)**, the existence of N_2_O production from grassland soils at extremely low air temperatures had to be extrapolated. The soil N_2_O production rates in thawing periods were significant higher than those in freezing periods, and mean N_2_O production from all soil cores during thawing periods averaged approximately three times more than that during freezing periods (**Tables 2 and 3**). This may indicate that microbial activity or population soared, and that enzyme activity increased when soils thawed at higher temperatures. Moreover, peaks in the N_2_O production rate were observed after soil thawing that lasted only a few days, and the production rate of N_2_O dropped off very quickly at the onset of freezing cycles (**S3 Fig**). The production rate also decreased during subsequent thawing periods (**Figs 3 and 4, Table 3**). Soil thawing enhanced microbial activity, resulting in higher oxygen consumption and induction of significant N_2_O emissions via greater denitrification activity [36, 62]. The soil NH_4_^+^ concentrations in the laboratory experiments were higher after freezing and thawing, indicating that there was net mineralization by microbial activity **(Fig 2)**. Increased soil NH_4_^+^ content would likely be supplied by soil organic matter [34, 63], and by the remains of destroyed microbes [56, 64]. Additionally, in the present study, we observed that, during a thawing period, higher production of N_2_O often occurred in the top of the soil column (0–6 cm), in contrast with that observed during a freezing period (**Figs 3 and 4**). Previous research indicated that most of the N_2_O emission was close to the soil surface rather than at depth in the soil profile [17, 65].

**Table 2:** Cumulative productions of N_2_O along the whole soil profile (0–15 cm) at different temperature during the freeze–thaw cycles of different land use/cover type^a^.

Plot code	Cumulative productions of N_2_O in incubation experiment (mg N_2_O-N kg^-1^)								
	−15°C	5°C	−10°C	5°C	−5°C	5°C	−5°C	10°C	Mean
UM	0.8±0.1^ABe^	12.9±1.1^Ba^	3.6±0.3 ^Bd^	10.6±0.8^Cb^	3.9±0.3 ^Bd^	12.3±0.8 ^Bab^	4.6±0.3 ^Bcd^	6.3±0.5 ^Bc^	6.9±0.5 ^B^
M1	0.8±0.1 ^ABe^	11.2±0.9 ^Bb^	4.3±0.3 ^Ad^	13.9±1.0 ^Ba^	4.5±0.3 ^Ad^	14.3±1.0 ^Aa^	6.1±0.4 ^Acd^	6.9±0.5 ^Bc^	7.8±0.6 ^B^
M3	0.9±0.1 ^Af^	27.6±1.9 ^Aa^	4.4±0.3 ^Ae^	21.7±1.4 ^Ab^	5.0±0.4 ^Ae^	13.1±0.9 ^Ac^	4.9±0.4 ^Be^	9.9±0.7 ^Ad^	10.9±0.7 ^A^
LUG	0.6±0.1 ^Bbc^	1.8±0.1 ^Da^	0.7±0.1 ^Dbc^	1.9±0.1 ^Da^	0.5±0.1 ^Cc^	0.7±0.1 ^Cbc^	0.6±0.1 ^Cbc^	0.8±0.1 ^Cb^	0.9±0.1 ^C^
SUG	1.0±0.1 ^Ad^	5.4±0.7 ^Ca^	2.1±0.2 ^Cc^	3.1±0.3^Db^	0.9±0.1 ^Cd^	1.2±0.2 ^Cd^	0.7±0.1 ^Cd^	1.1±0.1 ^Cd^	1.9±0.2 ^C^
SG	0.9±0.1 ^Abc^	2.1±0.2 ^Da^	1.0±0.1 ^Dbc^	2.1±0.2 ^Da^	0.8±0.1 ^Cc^	1.2±0.1 ^Cb^	0.7±0.1 ^Cc^	0.8±0.1 ^Cc^	1.2±0.1 ^C^

^a^The duration is 7 days at each temperature. Uppercase letters indicates significant differences (P<0.05) among different soil types. Lowercase letters indicates significant differences (P<0.05) among different temperature (mean ± SE, n = 3)

**Table 3:** Soil N_2_O production rates along the whole soil profile (0–15 cm) during the freeze–thaw cycles of different land use/cover type.

Plot code	Soil N_2_O production rates in incubation experiment (μg kg^-1^ h^-1^)							
	Cycle 1 −15~5°C	Cycle 2 5~−10°C	Cycle 3 −10~5°C	Cycle 4 5~−5°C	Cycle 5 −5~5°C	Cycle 6 5~ −5°C	Cycle 7 −5~10°C	Mean
UM	8.9±0.6^Ab^	8.9±0.7^Ba^	9.3±0.8^Ba^	9.4±0.7^Ca^	9.3±0.7^Ca^	9.4±0.7^Ca^	9.6±0.6^Aa^	8.4±0.8^B^
M1	6.0±1.6^Ac^	9.8±1.7^ABb^	11.7±0.5^Bab^	12.0±0.5^Bab^	11.7±0.4^Bab^	12.6±0.5^Ba^	12.7±0.8^Aa^	9.9±1.4^B^
M3	10.8±1.2^Ac^	12.2±1.2^Abc^	17.6±2.2^Aa^	17.3±1.3^Aa^	15.1±0.9^Aabc^	15.5±1.3^Aab^	11.6±0.8^Ac^	13.5±2.5^A^
LUG	2.2±0.4^Bab^	2.4±0.4^Da^	2.2±0.3^Dab^	1.6±0.1^Dbc^	1.4±0.1^Dc^	1.2±0.1^Dc^	1.2±0.1^Bc^	2.1±0.2^D^
SUG	3.2±0.3^Bbc^	5.5±1.5^Cab^	5.8±1.6^Ca^	2.5±0.3^Dc^	1.9±0.1^Dc^	1.7±0.1^Dc^	1.7±0.1^Bc^	4.3±0.8^C^
SG	2.8±0.3^Ba^	2.6±0.4^CDab^	2.6±0.5^CDab^	1.8±0.2^Dbc^	1.7±0.2^Dc^	1.5±0.2^Dc^	1.6±0.2^Bc^	2.5±0.2^D^

Uppercase letters indicates significant differences (P<0.05) among different soil types. Lowercase letters indicates significant differences (P<0.05) among different cycle (mean ± SE, n = 3)

Mean rates of N_2_O production ranged from 1.2 to 17.6 μg kg^-1^ h^-1^ for the entire soil profile (0–15 cm) during freezing and thawing periods (**Table 3**). Katayanagi and Hatano [29] reported that N_2_O emissions during a winter–spring period ranged from 0.48 to 6.63 μg kg^-1^ h^-1^. Those values are lower than our results (1.2 to 17.6 μg kg^-1^ h^-1^) because in the present study, the soil was bare, and there was no temperature-blocking effect in our laboratory study.

### Soil N_2_O flux from field measurement

**Fig 5:**
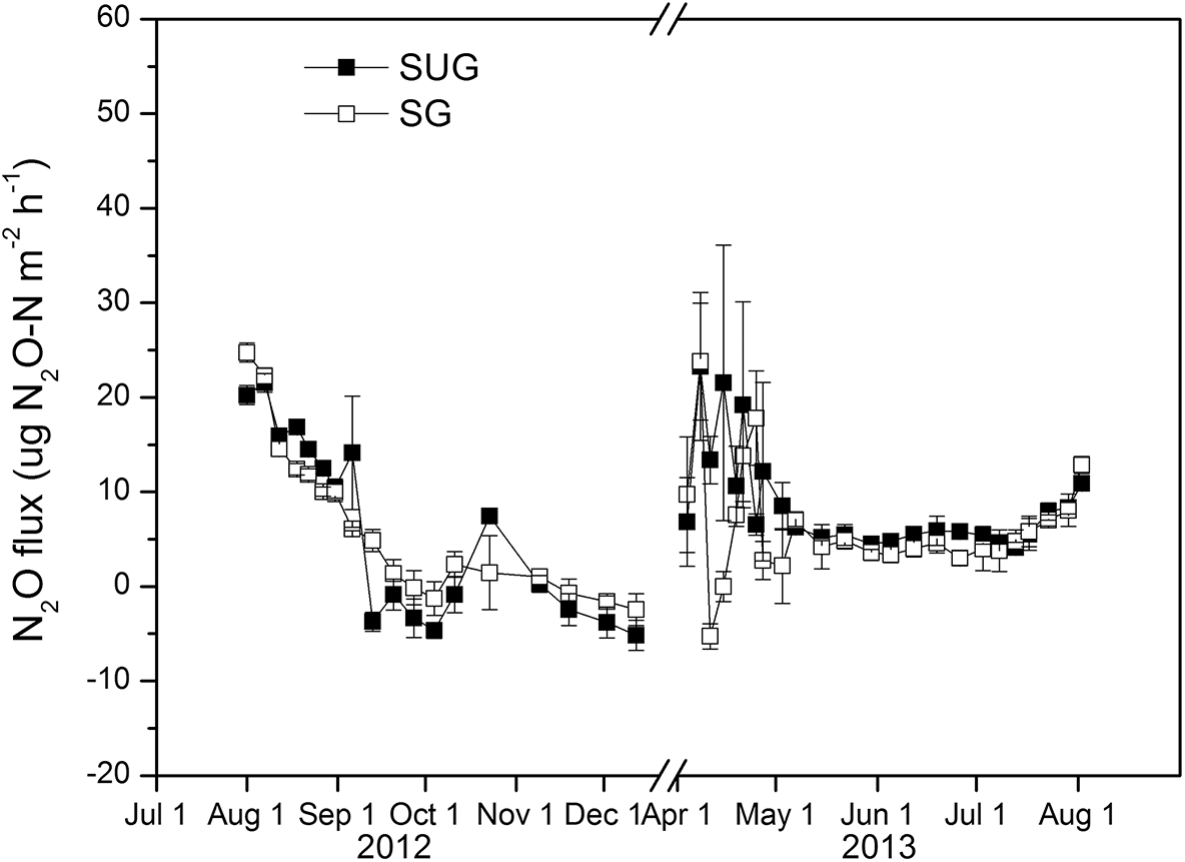
N_2_O emissions at the grazed (SG) and ungrazed (SUG) sites from August 2012 to August 2013 (mean ± SE, n = 4).

In our field measurements, N_2_O emissions during the freeze–thaw transition periods were much higher than observed for other periods (**Fig 5**). The increase in N_2_O emission flux was found during two phases of the FTCs: the progressive freezing period (from 16 October to 2 November 2012) and the progressive thawing period (from 28 March to 16 April 2013). This indicates that the FTCs had an important effect on N_2_O emissions. The higher N_2_O flux in the progressive thawing period was more likely to be ascribed to the heavy snow during the winter in 2012. Denmead et al. [65] reported that high N_2_O emission might be caused by the high moisture content during the freeze–thaw periods in the spring. Peaks in N_2_O emission occurred during highly favorable conditions, which included denitrification due to water-saturated topsoil [66], and high amounts of carbon and nitrogen available for the denitrification [67,68] as a result of the destruction of soil aggregates [60,68].

On the other hand, the SUG and SG plots also showed flux in N_2_O emissions—even if very low—during completely frozen periods (**Fig 5**). This is in line with the results from our laboratory experiment: some limited N_2_O production occurred even at extremely low air temperatures. The accumulation of N_2_O (0.17 and 0.09 μg N_2_O-N m^-2^ at SUG and SG plots, respectively) during the long completely frozen period (144 days) could be responsible for higher N_2_O flux at the beginning of the progressive thawing period. However, the measured values of N_2_O flux were sometimes negative. This agreed with the findings of Xu [69], who reported that negative fluxes in winter were found in the Xilin River basin of Inner Mongolia. This situation was also found in other terrestrial ecosystems (i.e., in temperate forest [70] and alpine grassland [71]), but the reasons why the soil absorbed N_2_O were not identified. Given a favorable environment with high precipitation and very little temperature increase in November, in our study (**Fig 1**), microbial activity increased. Thus, the negative N_2_O flux was likely due to microbial reduction of N_2_O to N_2_ through denitrification.

The Hulunber meadow steppes are a source of annual N_2_O emissions of 0.56 kg N_2_O-N ha^-1^ yr^-1^ and 0.40 kg N_2_O-N ha^-1^ yr^-1^ at SUG and SG plots, respectively. The results indicate that grazing reduced N_2_O emission by approximately 29%. The mean fluxes of N_2_O from in situ year-round measurements were 9.21±1.8 μg N_2_O-N m^-2^ h^-1^ (SUG) and 6.54±1.3 μg N_2_O-N m^-2^ h^-1^ (SG), which are much higher than those of the native and grazed LC steppes (4.4 and 3.4 μg N_2_O-N m^-2^ h^-1^, respectively) [72]. Holst et al. [5] noted that N_2_O fluxes were 8.2 (UG, un-grazed site) and 1.5 μg N_2_O m^-2^ h^-1^ (WG, winter-grazed site) during FTCs on an Inner Mongolian steppe. The average winter/spring fluxes were 7.37 (SUG) and 4.07 μg N_2_O m^-2^ h^-1^ (SG) in our field measurements (**Table 3**), and the N_2_O emission at SG was approximately three times greater than at the WG. This may be ascribed to the presence of more aboveground litter that provided more substrate for microbial activity and favorable conditions for denitrification, i.e., longer water retention time.

### Land use/cover effect on N_2_O production

The mean production of N_2_O was significantly lower at UM and M1, and higher at M3 (P < 0.05), but was not significant in FTC 1 (−15/5°C) and FTC 7 (−5/10°C). The N_2_O mean production rate was significantly lower at SG and LUG and higher at SUG in FTC 2 (5/−10°C) and FTC 3 (−10/5°C) (P < 0.05). The rates of N_2_O production were highest for M3 soils, intermediate for M1 and UM soils, and lowest for SUG, SG, and LUG soils (**Tables 2 and 3**). In general, incubation of soil cores revealed differences in freeze–thaw-induced N_2_O production between land use/cover types under similar temperature conditions (**Figs 3 and 4, Table 3**). This could be attributed to differences among the land use/cover types with respect to soil properties (i.e., soil organic matter, soil pH, C:N ratio, and the availability of inorganic N) [73, 74, 75]. On the other hand, N_2_O is emitted as intermediate product of complex biochemical processes of nitrification and denitrification in soil [76, 77], which is dependent on O_2_ and inorganic nitrogen (NH_4_^+^ and NO_3_^−^) availability. Our results showed that the SOM content declined at a mean rate of 0.06 g kg^-1^ d^-1^ and that inorganic concentration was enhanced at a mean rate of 0.20 mg kg^-1^ d^-1^ (**Fig 2**), which sustained nitrification and denitrification by soil microbes. Some biological processes are also significantly affected by soil pH [78, 79, 80]. For example, several denitrifying enzymes are sensitive to changes in pH, i.e., NO_3_^−^ reductase, NO_2_^−^ reductase and N_2_O reductase. Therefore, the alterations in microbes would potentially influence the N_2_O production rates during freeze–thaw periods.

The mean N_2_O production rates were 8.4±0.8 μg kg^-1^ h^-1^ at UM (*Leymus chinensis* and *Artemisia tanacetifolia*), 2.1±0.2 μg kg^-1^ h^-1^ at LUG (*Leymus chinensis*), and 4.3±0.8 μg kg^-1^ h^-1^ at SUG (*Stipa baicalensis*) in our laboratory study (**Table 3**). Obviously, there was a significant difference (P < 0.05) in N_2_O production intensity under different types of land cover (grassland vegetation types). Soil characteristics and grassland types are thought to follow complex interactions [81]. Moreover, plant exudates could provide sources of carbon and nitrogen, i.e., acids, sugars and enzymes [82], for microbe growth, and thus microbial communities are driven by the types of plant cover [83]. Plant types and exudates also influence abiotic factors. For example, Li [84] revealed that the concentrations of soil NH_4_^+^-N and NO_3_^**−**^-N would increase during the period of maximum grassland growth, and that the rate of N mineralization would therefore increase.

In our laboratory study, it was found that grazing decreased N_2_O production rates by 36.8% (mean 4.3±0.8 and 2.5±0.2 μg kg^-1^ h^-1^ at the SUG and SG plots, respectively) (P < 0.05) during the spring thaw (**Table 3**). In field measurements, grazing decreased the flux of N_2_O emission by 44.8% during the freeze–thaw period (mean 7.3±0.8 and 4.1±0.2 μg kg^-1^ h^-1^ at the SUG and SG plots, respectively). This was in agreement with the study by Wolf et al. [17], who concluded that winter grazing would reduce the soil microbial population, inorganic N, and water retention during winter. In our study, there was no significant difference in precipitation at the two sites (SUG and SG), but the wind had strong effects in this area during both winter and spring. During winter, some of the snow deposited on grazed grassland (SG) might melt or be blown away by winds, to a greater extent than on native grasslands (SUG). This is because of the presence of only a few and sparse clumps of standing dead grasses at SG sites [85]. On the other hand, the trampling of soil by animals produced conditions that favor denitrification (i.e., increased soil compaction and reduced soil aeration). Saggar et al. [86] reported that grazing could change the soil mineral-nitrogen content. Although there was insignificant effect from grazing on NH_4_^+^ and NO_3_^**−**^ content before incubation, the soil NO_3_^**−**^ content at SUG was higher than that at SG after incubation, because the conditions at the former provided more substrate for denitrification (**Fig 2**). In addition, the influence of summer grazing may also affect N_2_O emission through other processes.

In contrast, mowing increased N_2_O production at M3 sites (mean 13.5±2.5 μg kg^-1^ h^-1^) compared with that at M1 sites (mean 9.9±1.4 μg kg^-1^ h^-1^) and at UM sites (mean 8.4±0.8 μg kg^-1^ h^-1^) during freeze–thaw periods (P < 0.05) (**Table 3**). Our plot data showed that mowing provided a marginally significant effect in preventing grassland degradation. The dominant species of unmown grassland-meadow was *Carex tristachya*, in contrast with *Leymus chinensis* and *Artemisia tanacetifolia* of mown grasslands (**Table 1**). Thus, difference in vegetation type caused by mowing could affect N_2_O production. Furthermore, the supply of photosynthates to roots was reduced due to removal of the aboveground biomass, which thereby inhibited the growth of roots and their absorption of soil nutrients. Hence, mowing may influence soil physico-chemical properties, i.e., bulk density, pH, and inorganic nitrogen. Collins et al. [19] found that the species richness of mown sites was roughly double that of unmown sites, and that mowing in anthropogenically stressed grasslands enhanced biodiversity. Bahn et al. [21] and Zhou et al. [22] found that mowing strongly decreased R_H_ (soil CO_2_ efflux from heterotrophs) (P < 0.05), while there was insignificant effect on R_A_ (soil CO_2_ efflux from autotrophs). Consequently, we presumed that mowing might alter microbial activity.

In addition, **Table 3** shows that N_2_O production in mown areas was higher than that in grazed areas, and that there were significant differences between the mown and grazed areas in each FTC. Therefore, we could conclude that the effects of different land uses (grazing and mowing) on N_2_O production were different and cannot be neglected.

## Conclusions

Our results derived from laboratory and in situ experiments clearly showed that pulses of N_2_O emission from meadow-steppe soils follow freeze/thaw events. The increasing N_2_O was most likely due to production during thawing periods within the top soil (0–6 cm), while N_2_O production could also be found during the frozen periods, even at extremely low air temperature (minimum −37.8°C). There were different patterns and magnitudes of soil N_2_O flux during the winter–spring period across the investigated land uses (mowing versus grazing) and land covers (grassland vegetation). N_2_O production rate at the grazed site (SG, 2.5±0.2 μg kg^-1^ h^-1^) was lower than at the ungrazed site (SUG, 4.3±0.8 μg kg^-1^ h^-1^), and grazing decreased N_2_O production by 37% (1.2±0.1 versus 1.9±0.2 mg kg^-1^) during the whole freeze–thaw cycles. The N_2_O production rate was related to the rate at which grassland was mowed. The mean N_2_O production rates (μg kg^-1^ h^-1^) were as follows: triennially (M3, 13.5±2.5) > once annually (M1, 9.9±1.4) ≥ unmown (UM, 8.4±0.8), and N_2_O production at M3 were approx. 58% and 13% averaged higher than at UM. It is noteworthy that mowing and grazing should be differentiated rather than presumed to be equal, because they had opposite effects (enhancing versus reducing) on N_2_O emission. Significantly increased emissions of N_2_O were observed during FTCs in our year-round field measurements. The mean N_2_O fluxes were 9.21±1.8 and 6.54±1.3 μg N_2_O-N m^-2^ h^-1^ at SUG and SG sites, respectively, and grazing reduced N_2_O emission by approximately 29%.

## Supporting Information

S1 Fig Mean CO_2_ production rates (μg g^-1^ h^-1^) along the soil profile (0–15 cm) of the undisturbed soil cores: From UM (a, b), M1 (c, d), M3 (e, f) over the entire incubation period (n = 3); Freezing periods: a, c, e; Thawing period: b, d, f. (TIF)

S2 Fig Mean CO_2_ production rates (μg g^-1^ h^-1^) along the soil profile (0–15 cm) of the undisturbed soil cores: From LUG (a, b), SUG (c, d), SG (e, f) over the entire incubation period (n = 3); Freezing period: a, c, e; Thawing period: b, d, f. (TIF)

S3 Fig Dynamics of the N_2_O production rates (μg kg^-1^ h^-1^) along the soil profile (0–15 cm) of the undisturbed soil cores: From UM (a) M1(b) M3(c) LUG(d) SUG(e) SG(f) during the entire incubation period (n = 3). (TIF)

S4 Fig Correlation between CO_2_ and N_2_O production rates over the entire incubation period. (TIF)
