# Supplementary figures and images for: Influences of Land Use/Cover Types on Nitrous Oxide Emissions during Freeze-Thaw Periods from Waterlogged Soils in Inner Mongolia
Source: PLoS One. 2015 Sep 25;10(9):e0139316. doi: 10.1371/journal.pone.0139316 (PMC4583501; doi:10.1371/journal.pone.0139316)

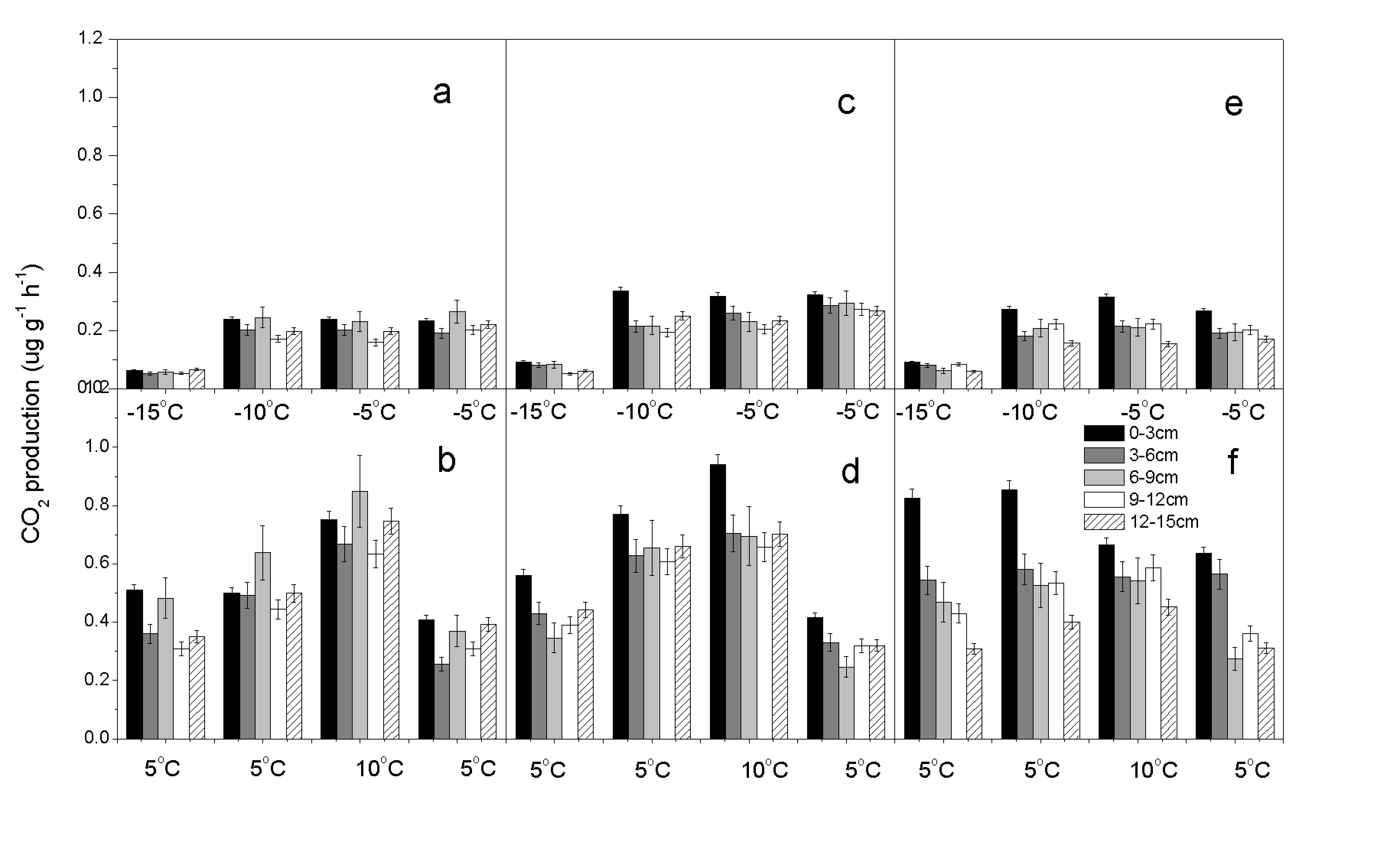

Supplement: S1 Fig — (TIF) [file pone.0139316.s001.tif]

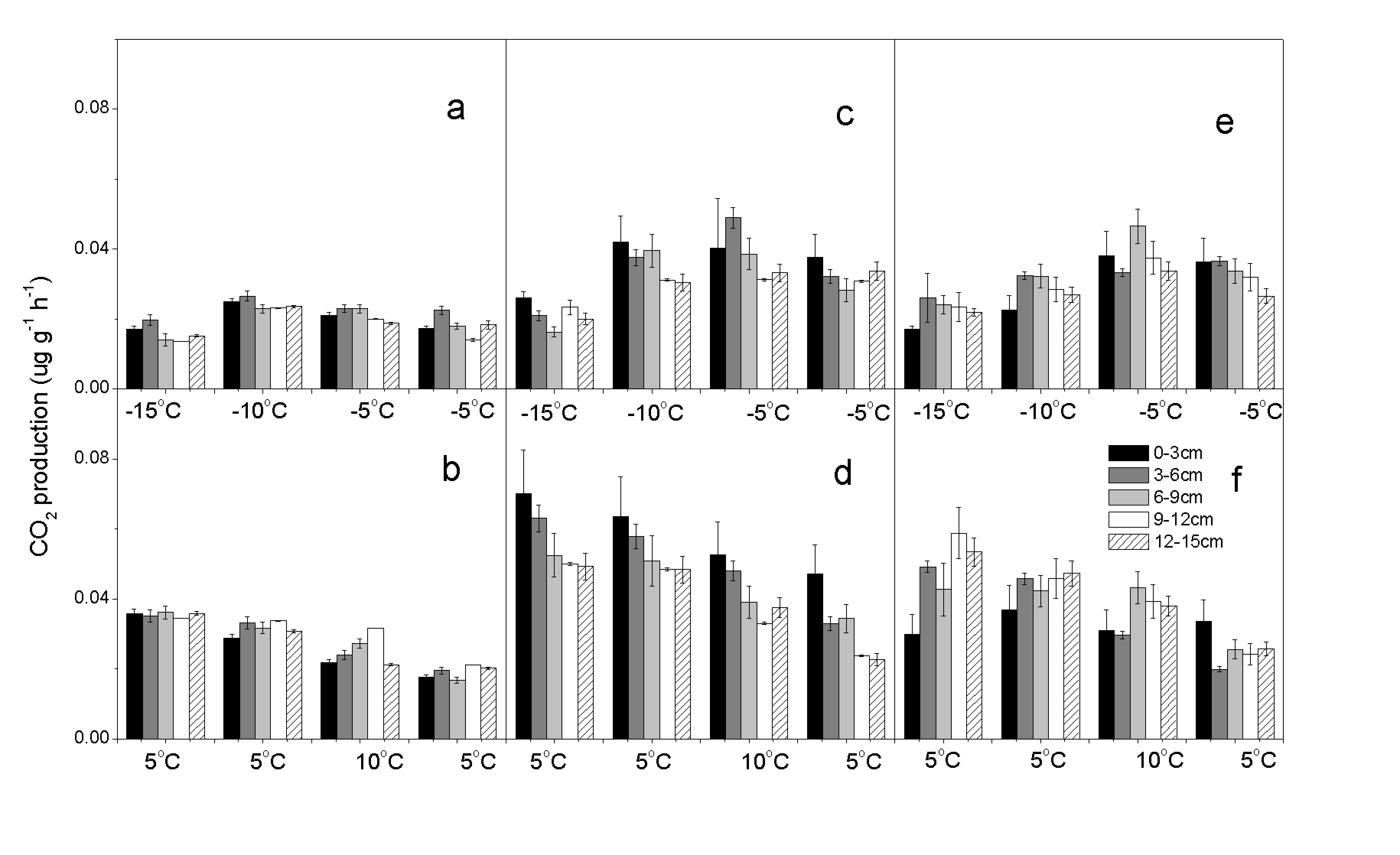

Supplement: S2 Fig — (TIF) [file pone.0139316.s002.tif]

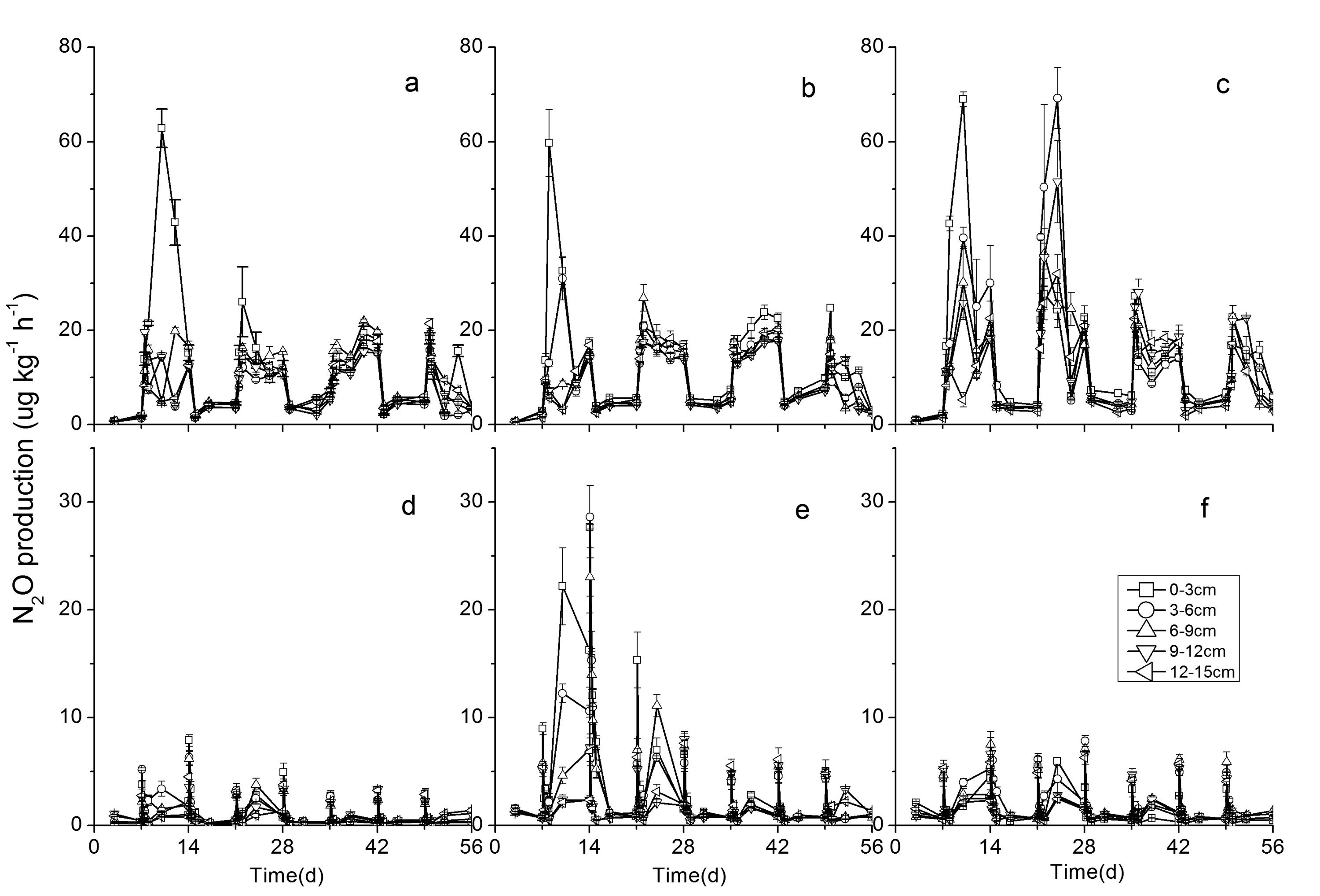

Supplement: S3 Fig — (TIF) [file pone.0139316.s003.tif]

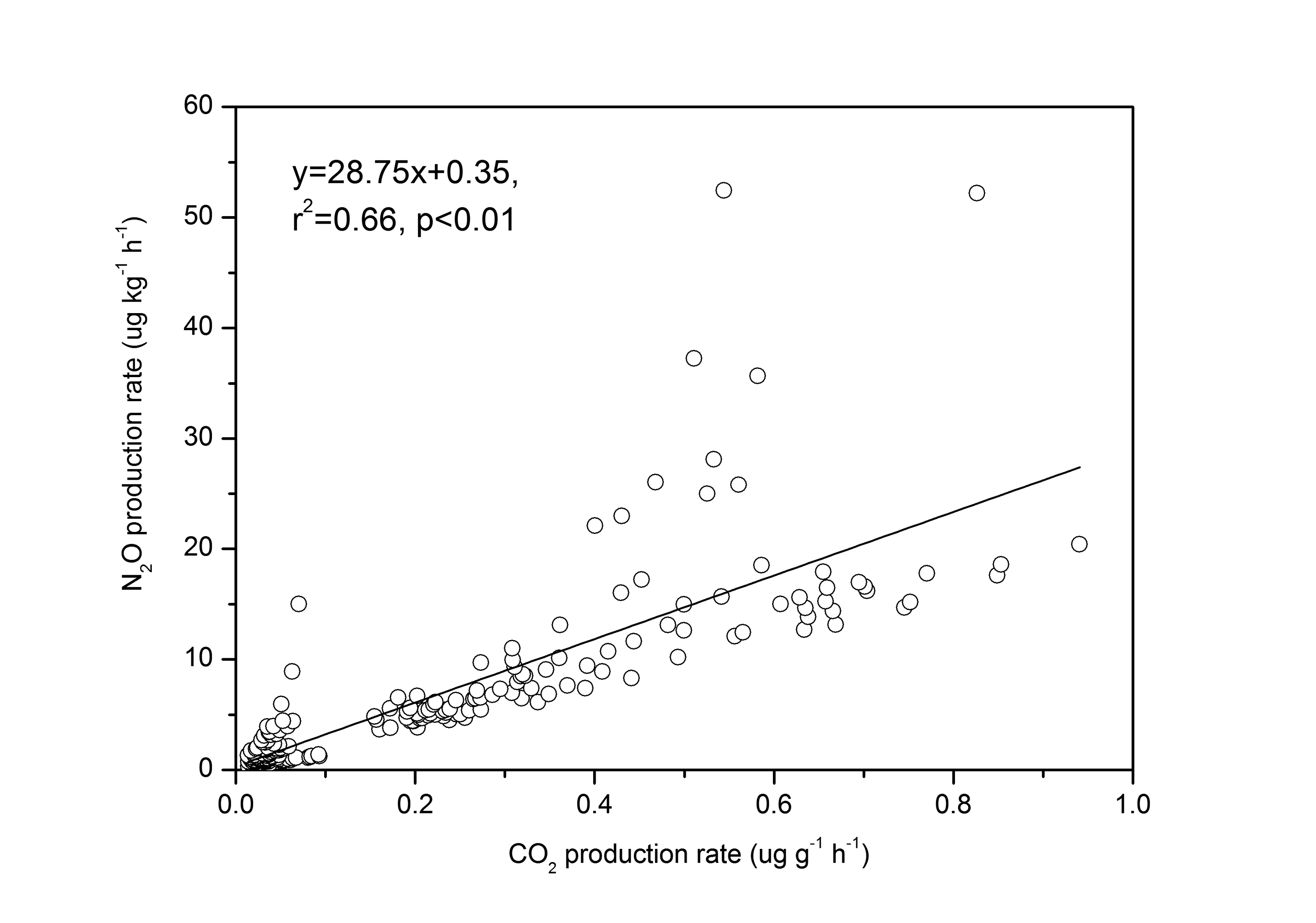

Supplement: S4 Fig — (TIF) [file pone.0139316.s004.tif]
